# Proteomic Analysis of the Effects of Aged Garlic Extract and Its FruArg Component on Lipopolysaccharide-Induced Neuroinflammatory Response in Microglial Cells

**DOI:** 10.1371/journal.pone.0113531

**Published:** 2014-11-24

**Authors:** Hui Zhou, Zhe Qu, Valeri V. Mossine, Dineo L. Nknolise, Jilong Li, Zhenzhou Chen, Jianlin Cheng, C. Michael Greenlief, Thomas P. Mawhinney, Paula N. Brown, Kevin L. Fritsche, Mark Hannink, Dennis B. Lubahn, Grace Y. Sun, Zezong Gu

**Affiliations:** 1 Department of Pathology and Anatomical Sciences, University of Missouri School of Medicine, Columbia, Missouri, United States of America; 2 Department of Biochemistry, University of Missouri School of Medicine, Columbia, Missouri, United States of America; 3 Center for Translational Neuroscience, University of Missouri School of Medicine, Columbia, Missouri, United States of America; 4 Department of Computer Science, Informatics Institute, University of Missouri, Columbia, Missouri, United States of America; 5 Department of Chemistry, University of Missouri, Columbia, Missouri, United States of America; 6 Division of Animal Sciences, University of Missouri, Columbia, Missouri, United States of America; 7 Harry S. Truman Veterans Hospital, Columbia, Missouri, United States of America; 8 British Columbia Institute of Technology, Vancouver, British Columbia, Canada; Carleton University, Canada

## Abstract

Aged garlic extract (AGE) is widely used as a dietary supplement, and is claimed to promote human health through anti-oxidant/anti-inflammatory activities with hypolipidemic, antiplatelet and neuroprotective effects. Prior studies of AGE have mainly focused on its organosulfur compounds, with little attention paid to its carbohydrate derivatives, such as *N*-α-(1-deoxy-D-fructos-1-yl)-L-arginine (FruArg). The goal of this study is to investigate actions of AGE and FruArg on antioxidative and neuroinflammatory responses in lipopolysaccharide (LPS)-activated murine BV-2 microglial cells using a proteomic approach. Our data show that both AGE and FruArg can significantly inhibit LPS-induced nitric oxide (NO) production in BV-2 cells. Quantitative proteomic analysis by combining two dimensional differential in-gel electrophoresis (2D-DIGE) with mass spectrometry revealed that expressions of 26 proteins were significantly altered upon LPS exposure, while levels of 20 and 21 proteins exhibited significant changes in response to AGE and FruArg treatments, respectively, in LPS-stimulated BV-2 cells. Notably, approximate 78% of the proteins responding to AGE and FruArg treatments are in common, suggesting that FruArg is a major active component of AGE. MULTICOM-PDCN and Ingenuity Pathway Analyses indicate that the proteins differentially affected by treatment with AGE and FruArg are involved in inflammatory responses and the Nrf2-mediated oxidative stress response. Collectively, these results suggest that AGE and FruArg attenuate neuroinflammatory responses and promote resilience in LPS-activated BV-2 cells by suppressing NO production and by regulating expression of multiple protein targets associated with oxidative stress.

## Introduction

Garlic is one of the most widely used botanicals worldwide, and studies have shown its beneficial effects against hypercholesterolemia and hypertension, and protection against cardiovascular disease and stroke [1]–[3]. Aged garlic extract (AGE), prepared from fresh garlic soaked in 15–20% aqueous ethanol and extracted/aged for more than 10 months at room temperature [4], has been demonstrated as a potent superoxide radical scavenger and chelator of transition metals [5]. While many studies have focused on the organosulfur compounds in AGE, including *S*-allyl-L-cysteine (SAC), allicin and allyl thiosulfinates [6], less attention has been paid to the carbohydrate derivatives, such as *N*-α-(1-deoxy-D-fructos-1-yl)-L-arginine (FruArg), as bioactive components [7],[8].

FruArg belongs to the class of fructosamines, which originate from a non-enzymatic reaction between glucose and arginine [9]; they modify proteins *in vivo* and are widely used as a diagnostic marker of long-term glucose concentration in diabetics. Fructosamine derivatives are formed in foods upon storage or dehydration and are regarded as a functional food [9]. There is evidence that fructose-amino acids can act as immune-stimulants and inhibit tumorigenesis and metastasis in animal models of cancer [10]–[13].

FruArg was initially extracted from Korean red ginseng as a novel substrate of nitric oxide synthase [14],[15]. It has been also identified as a major component in AGE and is generally present at 2–2.5 mM concentration [16]. FruArg exhibits antioxidant properties and is capable of scavenging hydrogen peroxide and protecting macrophages or endothelial cells from the damaging effects of oxidized low-density lipoprotein [16],[17]. *In vivo*, FruArg was shown to suppress noradrenalin-induced hypertension and reduce postprandial blood glucose level [18]. These findings suggest that AGE and FruArg may offer beneficial effects by reduction of chronic innate immune activation [19],[20]. As a part of our long-standing interest in dietary antioxidants in promotion of resilience in brain health, an important goal for this study is to investigate the protective effects of AGE and FruArg in neuroinflammation and elucidate their mode(s) of action in microglial cells.

Microglia are the resident immune effector cells in the central nervous system (CNS) with the ability to confer resilience against oxidative and inflammatory responses by increasing production of the anti-oxidative products in responding to various types of injuries and environmental stress [21]–[24]. Besides maintenance of immune response, microglial cells can be activated upon phagocytosis of invading bacteria or endocytosis of toxins and produce reactive oxygen/nitrogen species including nitric oxide (NO). Excessive production of NO can induce nitrosative stress in the cell and contributes to neurovascular injuries leading to neurodegenerative diseases including traumatic brain injury, cerebral ischemia, Parkinson's disease and Alzheimer's disease [24]–[29]. Therefore, agents that can attenuate microglial activation, suppress chronic production of proinflammatory molecules, and/or increase production of antioxidants in the brain are of interest for the development of novel approaches in prevention of neurodegenerative diseases [30],[31].

In the present study, we assessed effects of AGE and FruArg in lipopolysaccharide (LPS)-activated murine BV-2 microglial cells, a well-defined paradigm for study of neuroinflammatory responses. Quantitative proteomic analyses by two dimensional differential in-gel electrophoresis (2D-DIGE) combined with liquid chromatography tandem mass spectrometry (LC-MS/MS) identified multiple molecular targets of AGE and FruArg in LPS-stimulated BV-2 cells. Using Ingenuity Pathway Analysis (IPA) and MULTICOM-PDCN analysis, we predicted signal transduction pathways and protein networks that are modulated by AGE and FruArg, thus providing important insights into the molecular mechanisms that may underlie their beneficial effects in brain health and promotion of resilience.

## Materials and Methods

### Materials

**Figure 1:**
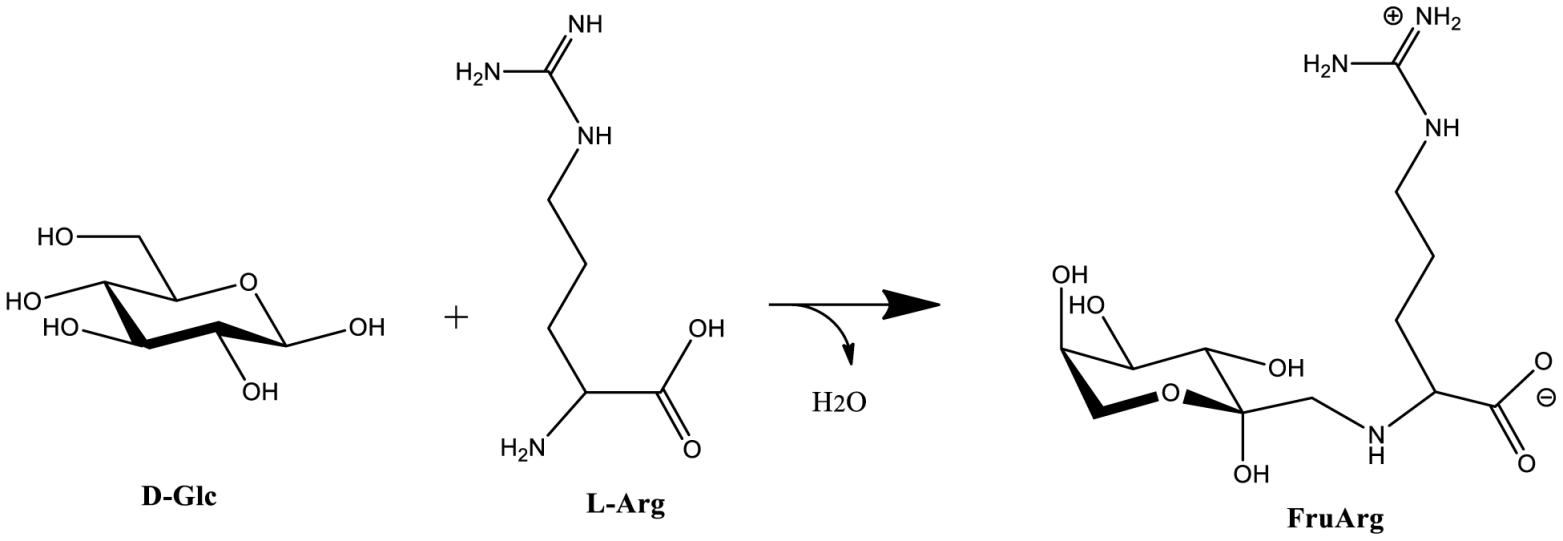
Structure and chemical reaction of FruArg. Condensation reaction between D-glucose and L-arginine, followed by the Amadori rearrangement, yields FruArg. FruArg is shown as a zwitterion and in the β-pyranose conformation, which are predominant forms at physiological pH.

Dulbecco's modified Eagle's medium (DMEM), penicillin-streptomycin and L-glutamine were obtained from Gibco (Grand Island, NY, USA). Fetal bovine serum (FBS) was purchased from Atlanta Biologicals, Inc. (Lawrenceville, GA, USA). Aqueous AGE was obtained from Wakunaga of America (Mission Viejo, CA, USA) with the Kyolic brand name, and its content of the benchmark component SAC measured (see the analysis below). FruArg was synthesized by refluxing L-arginine and D-glucose according to the published protocol (Figure 1) [32], and its purity and stability measured by thin layer chromatograph as previously described [14], ^1^H- and ^13^C NMR spectroscopy, as well as electrospray ionization mass spectrometry. Dithiothreitol (DTT), iodoacetamide, 2-mercaptoethanol, neocuproine, [3-(4,5-dimethylthiazol-2-yl)-2,5-diphenyl-2H-tetrazolium bromide (MTT), broad-spectrum NO synthase (NOS) inhibitor *N*-ω-nitro-L-arginine methyl ester hydrochloride (L-NAME), protein inhibitor cocktail, LPS (rough strains) from *Escherichia coli* F583 (Rd mutant) were purchased from Sigma-Aldrich (St. Louis, MO, USA). CyDye DIGE Fluor Minimal Labeling Kit, immobilized pH gradient (IPG) buffer (pH 3–10), and Immobiline DryStrip gels (24 cm, pH 3–10) were obtained from GE Healthcare Life Science (Buckinghamshire, UK). Trypsin (modified, sequencing grade) was obtained from Promega (Madison, WI, USA). The bicinchoninic acid (BCA) protein assay kit, peroxiredoxin-1 (PRDX1) antibody (PA3750), glutaredoxin-3 (GLRX3) antibody (PA531160), and alpha-enolase (ENO1) antibody (PA521387) were purchased from Thermo Fisher Scientific-Pierce (Rockford, IL, USA). Caspase-1 (CASP1) antibody (SC-56036) was purchased from Santa Cruz Biotechnology (Santa Cruz, CA, USA).

### Analysis of AGE

The dry matter content of the AGE used in this study was 30.9%. SAC concentration in the AGE sample was determined to be 1652.05±0.08 µg/mL based on analysis of triplicates, determined by HPLC with a Primary Grade Botanical Reference Material (BRM) SAC (Chromadex Inc., Irvine, CA, USA) as an external calibration standard to comply with the identity testing requirements of cGMP (current Good Manufacturing Practices). Chromatographic separation of AGE was achieved on a C18, 4.6×100 mm, 2.6 µm column at 36°C. The injection volume was 5 µL with a flow rate of 1.0 mL/min. Mobile Phase A consisted of water with 20 mM sodium dihydrogen phosphate+10 mM heptane sulfonic acid, pH 2.1 and Mobile Phase B consisted of acetonitrile (ACN):water with 20 mM sodium dihydrogen phosphate+10 mM heptane sulfonic acid, pH 2.1 (50∶50, v/v). A gradient program was used for separation: 100% Mobile Phase A to 70% Mobile Phase A over 8.0 min, 70% Mobile Phase A to 46% Mobile Phase A over 12.0 min, 46% Mobile Phase A to 0% Mobile Phase A over 1.0 min, hold at 0% Mobile Phase A for 2.0 min. Detection was achieved at 208 nm. An in-house validation study was performed to evaluate the method's linear range, method detection limit, limit of quantification, precision and recovery. Calibration curves for SAC were linear over the range of 0.5–50 µg/mL. The detection limit and limit of quantification for SAC were determined to be 0.11 µg/mL and 0.20 µg/mL, respectively, and the overall average recovery and relative standard deviation was 100.3%±0.21%.

### Cell culture and treatment

Murine BV-2 microglial cells [33],[34] were received from co-author Dr. Grace Sun as a gift. The cells were cultured in DMEM containing 5% (v/v) heat-inactivated FBS, 25 U/mL penicillin, and 25 µg/mL streptomycin at 37°C in a saturated humidity atmosphere containing 95% (v/v) air and 5% (v/v) CO_2_ as previously described [35],[36]. At 70–80% confluence, cells were cultured in DMEM without serum for 4 h and then exposed to 100 ng/mL LPS for 20 h in the presence or absence of AGE (0.5%, v/v) and FruArg (3 mM), which were added to the culture medium 1 h prior to LPS exposure. As a positive control, 0.5 mM L-NAME was added to the medium 1 h prior to LPS exposure.

### Measurement of NO

NO production was evaluated by the Griess reaction: cell culture supernatants were mixed with an equal volume of Griess reagent [1% (w/v) sulfanilamide and 0.1% (w/v) *N*-(1-naphthyl) ethylenediamide in 5% (v/v) phosphoric acid] for 10 min at room temperature. Absorbance was measured at 543 nm using a Synergy-4 microplate reader (BioTek Instruments Inc., Winooski, VT, USA). NO concentration was determined from a standard curve of serial concentrations of sodium nitrite (0–100 µM).

### Cell viability assay

After treatment of BV-2 cells, the conditioned medium was replaced with DMEM containing 0.5 mg/mL MTT and incubated for 4 h at 37°C. The formazan products were dissolved in dimethyl sulfoxide (DMSO) and absorbance was measured at 540 nm using the Synergy-4 microplate reader.

### Labeling of proteins with CyDye reagents and gel fractionation

Cells were lysed in HENTS [250 mM HEPES-NaOH, pH 7.4, 1 mM EDTA, 0.1 mM neocuproine, 1% (v/v) Triton X-100, 0.1% (w/v) SDS] buffer containing 1% (v/v) protein inhibitor cocktail. Proteins were precipitated in 4× volume (v/v) acetone at −20°C overnight and then redissolved in lysis buffer (30 mM Tris-HCl, 7 M urea, 2 M thiourea, 4% (w/v) CHAPS, pH 8.5). After centrifugation at 15,000 g for 10 min, the supernatant was collected and protein concentration was determined using the BCA protein assay.

Twelve samples were generated from three biological replicates of four experimental conditions (untreated, LPS-treated, LPS+AGE and LPS+FruArg). Each sample was labeled with CyDye DIGE Fluor minimal dyes according to the manufacturer's protocol (ratio of 50 µg protein to 400 pmol CyDye dyes). To limit potential dye-specific labeling-derived artifacts, sample replicates in different gels were labeled with either Cy3 or Cy5 dye, whereas the internal standard pooled from all samples in equal amounts was labeled with Cy2 dye.

For two dimensional gel fractionation, samples labeled by CyDye dyes were pooled (150 µg protein total) at a 1∶1∶1 ratio and brought up to a final volume of 420 µL with rehydration buffer [7 M urea, 2 M thiourea, 4% (w/v) CHAPS, 2% (v/v) IPG buffer (pH 3–10), 0.002% (v/v) bromphenol blue, 2% (v/v) glycerol, 2% (v/v) 2-hydroxyethyl disulfide, 10 mM DTT]. The first-dimension isoelectric focusing (IEF) was performed in an IPGphor IEF unit (Bio-Rad, Berkeley, CA, USA) on 24-cm IPG strips pH 3–10 as follows: 30 V for 16 h, 250 V for 1 h, 1,000 V for 0.5 h and 8,000 V for up to a total of 80,000 Volt-hour. After IEF, the proteins in strips were reduced and alkylated by successive 15 min treatments with equilibration buffer containing 1.0% (w/v) DTT, followed by 2.5% (w/v) iodoacetamide. The proteins were then resolved in 12% SDS-PAGE gels casted in an Ettan DALTsix gradient maker (GE Healthcare Life Science, Buckinghamshire, UK).

### Gel image acquisition and 2D-DIGE data analysis

Fluorescence images of the 2D-DIGE gels were acquired using an Ettan DIGE imager scanner (GE Healthcare Life Science, Buckinghamshire, UK). Excitation and emission wavelengths were set specifically for each dye according to the manufacturer's recommendations. Semi-automated DIGE data analysis was then performed using the SameSpots software (Version 4.5; Totallab Nonlinear USA Inc. Durham, NC; http://www.totallab.com/products/samespots/overview/) [37]. We considered the fluorescence intensity changes as statistically significant when ratio >1.3 and p value<0.05 in one-way ANOVA analysis of the multiple sample groups.

### Protein identification by LC-MS/MS

After imaging for CyDyes, the gels were stained with Coomassie blue dye G250 following standard procedures. The gel spots of interest were manually picked and proteins were in-gel digested by trypsin. Briefly, gel slices were rinsed with 50 mM ammonium bicarbonate (NH_4_HCO_3_) and 50% (v/v) ACN, dehydrated with ACN and incubated in 50 mM NH_4_HCO_3_ containing 20 µg/mL trypsin (modified sequencing grade) on ice for 1 h and then at 37°C for 18 h. The tryptic peptides were extracted with three changes of the extraction solution containing 60% (v/v) ACN and 1% (v/v) trifluoroacetic acid. The solution containing peptides was evaporated to dryness and the dry peptide mixtures were dissolved in 5% (v/v) ACN/1% (v/v) formic acid.

Protein identification was performed with the Thermo Scientific LTQ Orbitrap-XL in positive ion reflector mode. A portion of the peptide digest (5 µL) was loaded onto a C8 trap column (C8 CapTrap; Michrom Bioresources, Auburn, CA). Bound peptides were eluted from this trap column onto a 10.5-cm long, 150-µm inner diameter, pulled-needle analytical column packed with Magic C18 reversed phase resin (Michrom Bioresources, Inc., Auburn, CA). The peptides were separated and eluted from the analytical column with a gradient of ACN at 400 nL/min as follows: initial conditions (during trap load) is 5% B (A: 0.1% formic acid in water; B: 99.9% ACN, 0.1% formic acid), ramp and hold at 10% B for 2 min, gradient from 10% to 40% B over 15 min, ramp to 90% B over 1 min, hold at 90% B for 11 min, ramp back to 5% B over 1 min, hold at 5% B for 5 min prior to loading next sample. The Proxeon Easy nLC system is attached to a LTQ Orbitrap-XL mass spectrometer. Following a high-resolution (30,000 res, profile) Fourier transform MS scan of the eluting peptides (300–2000 m/z range), in each cycle, the 9 most abundant peptides (reject trypsin autolysis ions) were subjected to ion-trap collision-induced dissociation (CID) peptide fragmentation (>1000 counts, NCE of 35%, centroid). Data across a total of 35 min of elution were collected. The Sorcerer 2IDA (Sage-N research Inc., San Jose, CA) was used to query the data against the IPI-mouse database. Criteria for database search were 25 ppm mass error, monoisotopic masses, and methionine oxidation as variable modification.

### Bioinformatics analysis

The identified proteins were analyzed by IPA software version 7.6 (http://www.ingenuity.com; Ingenuity Systems, Redwood City, CA, USA) for associated biological functions and molecular pathways, as well as to predict protein-protein interaction networks determined by the Ingenuity Knowledge Base [38], a structured repository of expertly curated biological interactions and functional annotations from various sources including the literature. Our in-house MULTICOM-PDCN software [39] was used to predict protein subcellular locations by searching the Swiss-Prot database.

## Results

### AGE and FruArg suppress NO production in LPS-stimulated BV-2 microglial cells

**Figure 2:**
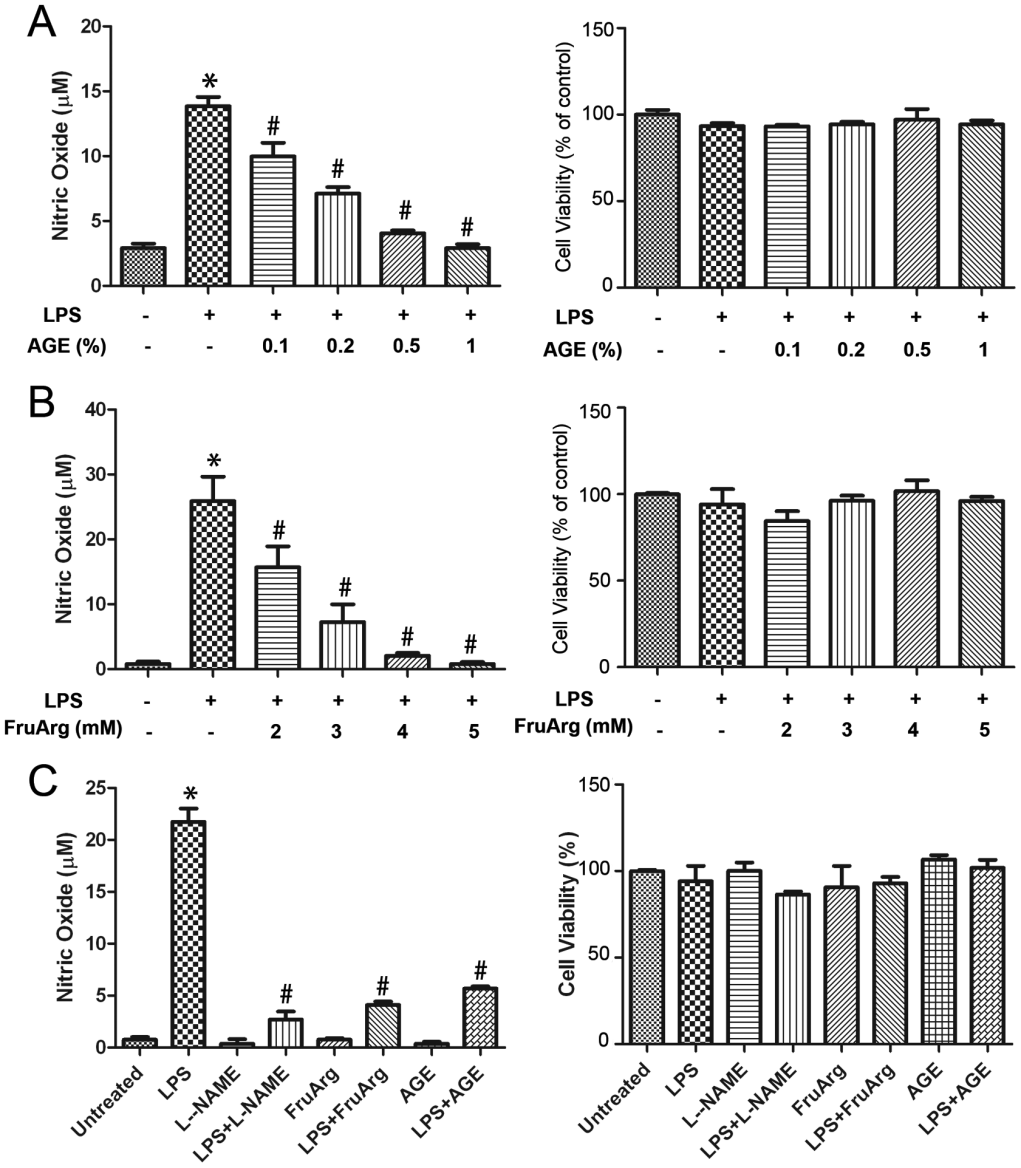
Effects of AGE and FruArg on NO production induced by LPS in BV-2 cells. (**A**) BV-2 cells were treated with LPS in the presence of AGE (0, 0.1%, 0.2%, 0.5%, or 1%) for 20 h. Griess assay (left plot) showed NO level significantly increased after LPS-exposure (*, p<0.05 vs. untreated), but was inhibited by AGE in a dose-dependent manner (#, p<0.05 vs. LPS-treated by one-way ANOVA, n = 3; data are presented as mean±SEM). Result of MTT assay indicated the addition of AGE did not significantly affect the cell viability (right plot). (**B**) Dose titration for administration of FruArg. BV-2 cells were exposed to LPS in the presence of various concentration of FruArg (0, 2, 3, 4, or 5 mM) for 20 h. LPS-induced NO production was significantly suppressed by FruArg in a dose-dependent manner (left plot; *, p<0.05 vs. untreated; #, p<0.05 vs. LPS-treated by one-way ANOVA, n = 3; data are presented as mean±SEM), however, the cell viability was not affected (right plot). (**C**) Effects of AGE and FruArg in LPS-stimulated BV-2 cells. According to above results, 0.5% for AGE and 3 mM for FruArg were selected as the treatment concentrations used for further study, respectively. Under such conditions, AGE and FruArg alone neither show any effect on NO production (left) nor on cell viability (right). L-NAME (0.5 mM), a known broad-spectrum NOS inhibitor, was introduced as a positive control. *, p<0.05 vs. untreated; #, p<0.05, vs. LPS-treated by one-way ANOVA, n = 3; Data are presented as mean±SEM.

As shown in Figure 2A (left plot), LPS stimulation significantly increased NO level in BV-2 microglial cells. Treatment of BV-2 cells with various concentrations of AGE (0.1%, 0.2%, 0.5%, and 1%) or FruArg (2, 3, 4, and 5 mM) in the presence of LPS suppressed NO levels in a concentration-dependent manner (Figure 2A, left plot). No significant cell death was observed by the MTT assay (Figure 2A, right plot). Similarly, FruArg attenuated LPS-induced NO production in a dose-dependent manner (Figure 2B, left plot) without affecting cell viability (Figure 2B, right plot). Based on these findings, we chose to use 0.5% AGE and 3 mM FruArg concentrations for proteomic analysis described below. At these concentrations, the treatments alone were not detectably cytotoxic nor showed any significant effects on NO production (Figure 2C). As a positive control, treatment with a known broad-spectrum NOS inhibitor L-NAME inhibited response in the LPS-stimulated BV-2 cells (Figure 2C, left plot).

### Proteomic analysis of AGE and FruArg treatment in LPS-stimulated BV-2 cells

**Figure 3:**
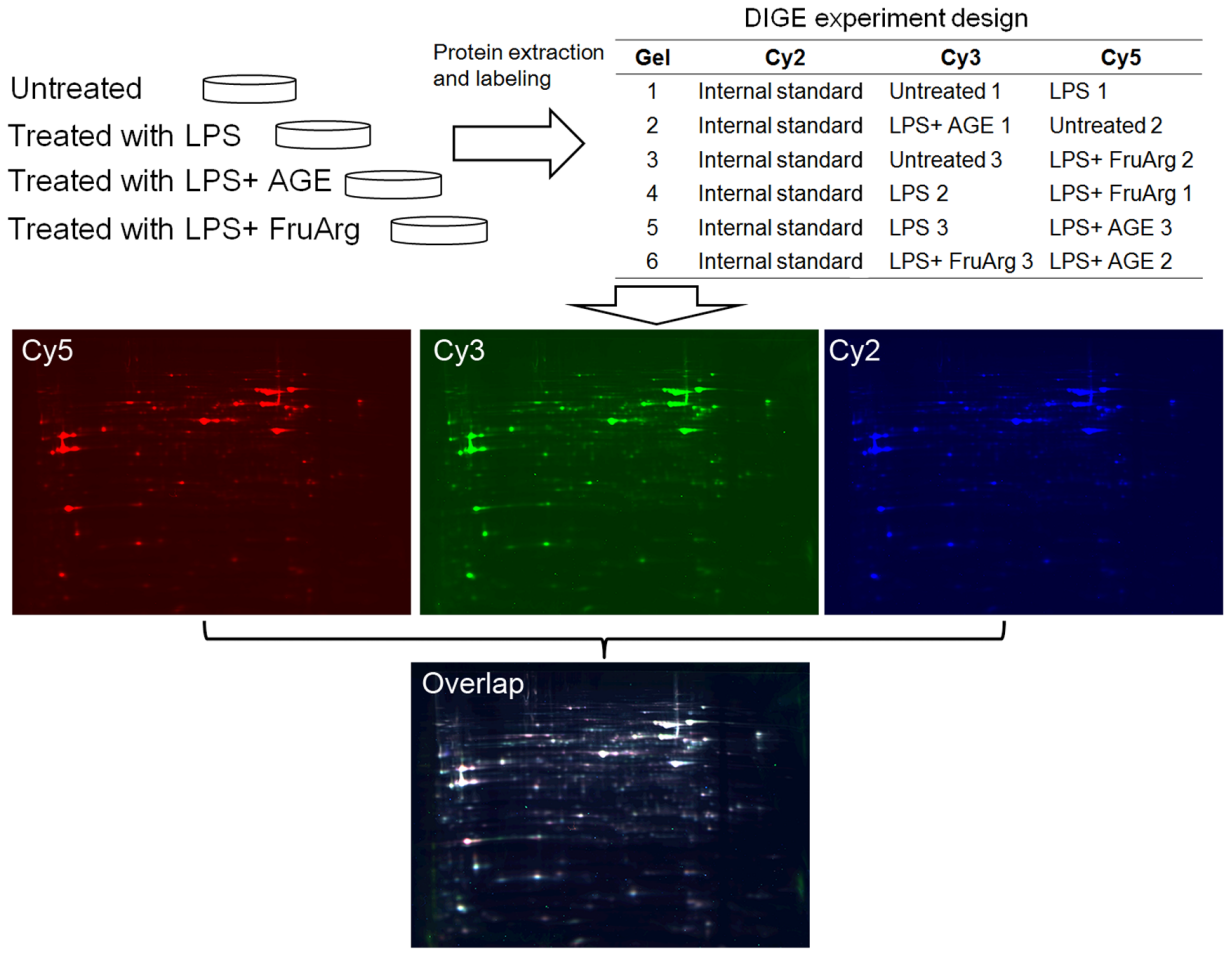
2D-DIGE experimental design and workflow. Lysates of BV-2 cells untreated or treated with LPS in the absence or presence of AGE (0.5%) or FruArg (3 mM) in triplicate were labeled with fluorescence CyDyes following the scheme shown in the table. The samples were then mixed and resolved on six independent 2D-DIGE gels. Three fluorescence images were obtained from each gel and subjected to image analysis using the SameSpots software.

**Figure 4:**
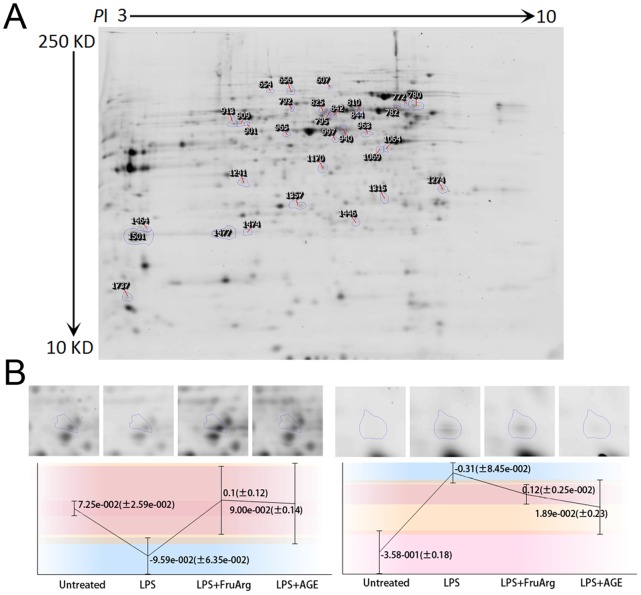
Differential spots detection by the SameSpots software. A total of 1,925 protein spots on the 24-cm 2D-DIGE gels were detected using SameSpots analysis. Compared to untreated conditions, 25 protein spots showed significant protein level changes (ratio >1.3, p<0.05) after treatment with LPS. In comparison to LPS treatment, 20 and 21 protein spots were altered by AGE and FruArg, respectively. All of these differential spots were indicated on a representative 2D-DIGE gel (**A**). Quantification results of two example spots, #825 (left) and #1737 (right), were shown (**B**).

Cell lysates from four experimental conditions (untreated, LPS-treated, LPS+AGE, and LPS+FruArg) in biological triplicates were labeled by three fluorescence CyDyes (Cy2, Cy3 and Cy5) (Figure 3). After 2D-DIGE separation, 1,925 protein spots on 24-cm gels were detected, of which 32 were significantly altered (ratio >1.3, p<0.05) by the treatments. A representative 2D-DIGE gel image is shown in Figure 4A; quantitative results of spot #825 (Figure 4B, left panel) and spot #1737 (Figure 4B, right panel) are presented as examples of up-regulation and down-regulation, respectively, in the treatment groups. The 32 spots with differential ratio changes were excised from the corresponding Coomassie Blue stained gel for protein identification by LC-MS/MS.

### Identification of differentially expressed proteins

**Figure 5:**
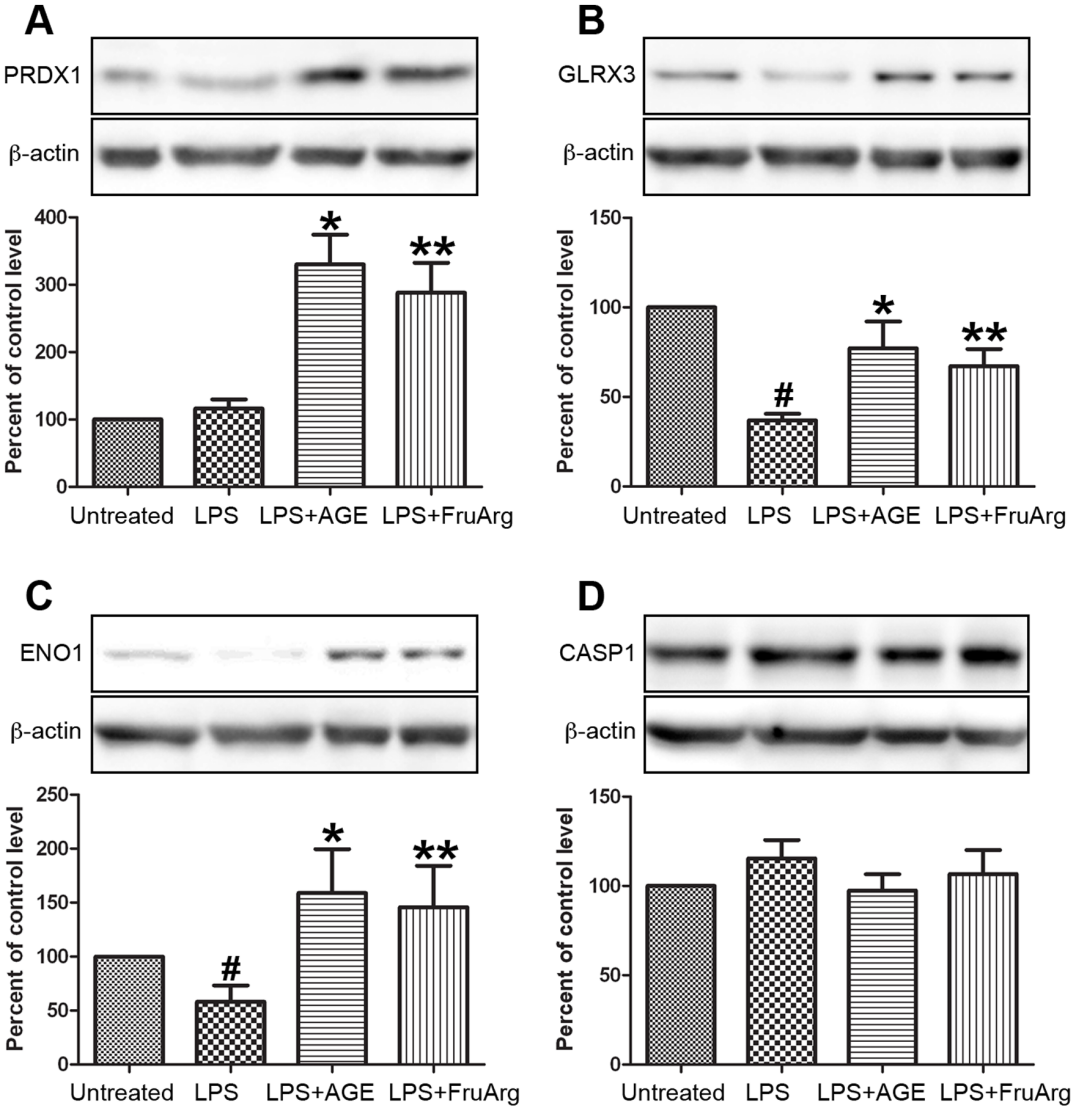
Validation of expression profiling of proteins by Western blotting. Four of the identified proteins, PRDX1 (**A**), GLRX3 (**B**), ENO1 (**C**), and CASP1 (**D**), responding to AGE and/or FruArg treatment in LPS-stimulated BV-2 cells were validated using Western blotting. Protein intensities were quantified by Image J software, normalized to β-actin, and expressed as percentage of untreated controls. Data are means ± SEM (n≥5); #, P<0.05, untreated vs. LPS; *, P<0.05, LPS vs. LPS+AGE; **, P<0.05, LPS vs. LPS+FruArg.

Results of protein identification using LC-MS/MS were listed in Table 1. Some of the protein spots contained more than one protein. For example, two proteins were found in spot #940 and recognized as ENO1 and CASP1. These proteins were identified with high confidence with sequence coverage in the range of 40% to 80%. In addition, some spots contained the same proteins, for instance, spots #1064 and #1069 were both identified as protein GLRX3 and PSMD13, suggesting the co-existence of their post-translational modifications or isoforms. Four of the identified proteins were selected for validation using Western blotting (Figure 5). The results showed that PRDX1 could be up-regulated by both AGE and FruArg (Figure 5A), while GLRX3 was down-regulated by LPS and up-regulated by AGE and FruArg (Figure 5B). ENO1 decreased responding to LPS but increased after AGE or FruArg treatment (Figure 5C), whereas CASP1 did not show significant changes under different treatment conditions (Figure 5D), suggesting that ENO1 mainly contributed to the changes in spot #940.

**Table 1:** List of the differentially expressed proteins identified by 2D-DIGE and LC-MS/MS analysis.

Spot number^a^	Ratios^b^			Protein accession	Protein name	Symbol	Molecular weight (Da)	Number of unique peptides	Number of unique spectra	Number of total spectra	Sequence coverage
	LPS/untreated	LPS+AGE/LPS	LPS+FruArg/LPS								
607	−1.69	2.04	1.68	IPI00330862	Ezrin	EZR	69,408.80	31	41	44	47.40%
654	−1.38	−1.22	1.18	IPI00230035	ATP-dependent RNA helicase DDX3X	ERH	73,103.70	56	90	156	65.10%
656	−1.25	1.55	1.77	IPI00110588	Moesin	MSN	67,768.80	56	99	163	73.10%
772	−1.84	1.82	2.35	IPI00403810	Tubulin alpha-1C chain	TUBA1C	49,909.60	23	43	140	58.10%
780	1.27	1.2	1.37	IPI00310091	Serine/threonine-protein phosphatase 2A 65 kDa regulatory subunit A alpha isoform	PPP2R1A	65,323.70	37	67	142	62.80%
782	−1.47	−1.03	1.1	IPI00223253, IPI00224575, IPI00890005	Isoform 1 of Heterogeneous nuclear ribonucleoprotein K	HNRNPK	51,030.40	22	41	58	56.40%
792	1.41	3.03	1.95	IPI00126083	EH domain-containing protein 1	EHD1	60,605.20	41	70	99	79.80%
795	−1.39	1.58	1.41	IPI00228385	Glucose-6-phosphate 1-dehydrogenase X	G6PD	59,263.60	39	59	89	64.50%
				IPI00323600	Coronin-1A	CORO1A	50,988.90	29	47	88	53.40%
810	1.69	−1.96	−1.38	IPI00230108	Protein disulfide-isomerase A3	PDIA3	56,680.40	50	70	107	74.90%
825	−1.47	1.55	1.58	IPI00228385	Glucose-6-phosphate 1-dehydrogenase X	G6PD	59,263.60	44	81	271	77.90%
842	−1.19	1.45	1.5	IPI00225961	D-3-phosphoglycerate dehydrogenase	PHGDH	56,585.20	33	50	114	54.60%
844	−1.48	1.41	1.52	IPI00230139	Peptidyl-prolyl cis-trans isomerase FKBP4	FKBP4	51,573.10	46	84	119	76.00%
901	2.69	1.14	1.06	IPI00111285	Immune-responsive gene 1 protein	IRG1	53,759.60	30	55	104	67.60%
909	2.2	1.39	1.21	IPI00111285	Immune-responsive gene 1 protein	IRG1	53,759.60	32	62	159	68.90%
913	2.64	1.41	1.27	IPI00111285	Immune-responsive gene 1 protein	IRG1	53,759.60	30	57	151	68.90%
940	1.08	1.23	1.49	IPI00125676	Caspase-1	CASP1	45,642.00	27	46	62	53.00%
				IPI00462072	Alpha-enolase	ENO1	47,141.70	24	40	61	44.50%
963	2.22	−1.83	−1.09	IPI00133417	Interferon-induced protein with tetratricopeptide repeats 3	IFIT3	47,223.80	44	79	153	83.60%
965	−2.17	1.32	1.43	IPI00111013	Cathepsin D	CTSD	44,955.00	22	38	48	52.40%
997	3.82	−1.87	−1.54	IPI00120113	UMP-CMP kinase 2, mitochondrial	CMPK2	50,036.40	37	59	188	73.20%
1064	1.29	1.42	1.33	IPI00315550	Glutaredoxin-3	GLRX3	37,778.70	24	44	80	53.40%
				IPI00126048	26S proteasome non-ATPase regulatory subunit 13	PSMD13	42,810.00	23	31	36	54.50%
1069	−2.38	1.58	1.34	IPI00315550	Glutaredoxin-3	GLRX3	37,778.70	23	39	86	52.20%
				IPI00126048	26S proteasome non-ATPase regulatory subunit 13	PSMD13	42,810.00	25	37	45	61.70%
1170	1.13	1.48	1.35	IPI00314950	60S acidic ribosomal protein P0	RPLP0	34,217.50	24	42	136	69.70%
1241	−2.06	−1.08	−1.04	IPI00122547	Voltage-dependent anion-selective channel protein 2	VDAC2	31,733.60	16	22	78	55.90%
1274	−1.42	1.02	−1.33	IPI00230044	Isoform 2 of Tropomyosin alpha-3 chain	TPM3	29,021.20	35	67	165	77.40%
1315	1.38	−1.03	1.42	IPI00114329	Glutamate–cysteine ligase regulatory subunit	GCLM	30,535.20	15	23	46	50.00%
1357	1.38	1.13	1.06	IPI00457898	Phosphoglycerate mutase 1	PGAM1	28,832.80	22	53	112	78.70%
1446	1.48	−1.03	1.13	IPI00126042	Ras-related protein Rab-14	RAB14	23,897.60	18	23	29	64.70%
				IPI00132397	GTP-binding protein SAR1b	SAR1B	22,382.90	12	22	32	60.60%
				IPI00117264, IPI00895414	Protein DJ-1	PARK7	19,908.10	12	16	24	60.80%
1464	1.43	1.28	1.21	IPI00555023	Glutathione S-transferase P 1	GSTP1	23,610.10	10	17	52	48.60%
1474	2.78	−1.76	−1.35	IPI00121788	Peroxiredoxin-1	PRDX1	22,177.50	18	24	39	64.30%
				IPI00109109	Superoxide dismutase [Mn], mitochondrial	SOD2	24,603.20	12	17	29	44.60%
1477	2.35	−1.2	1.04	IPI00121788	Peroxiredoxin-1	PRDX1	22,177.50	24	43	131	76.40%
				IPI00109109	Superoxide dismutase [Mn], mitochondrial	SOD2	24,603.20	15	28	113	44.60%
1501	1.01	1.34	1.62	IPI00121788	Peroxiredoxin-1	PRDX1	22,177.50	26	52	310	80.40%
1737	4.57	−1.89	−1.52	IPI00555085	Ubiquitin-like protein ISG15	ISG15	17,897.50	9	21	60	55.30%

a. Protein spot number according to Figure 4.

b. Average volume ratio based on the spots normalized volume across the four groups was calculated by the SameSpots software. A *p* value for the one-way analysis of variance <0.05 was accepted.

**Figure 6:**
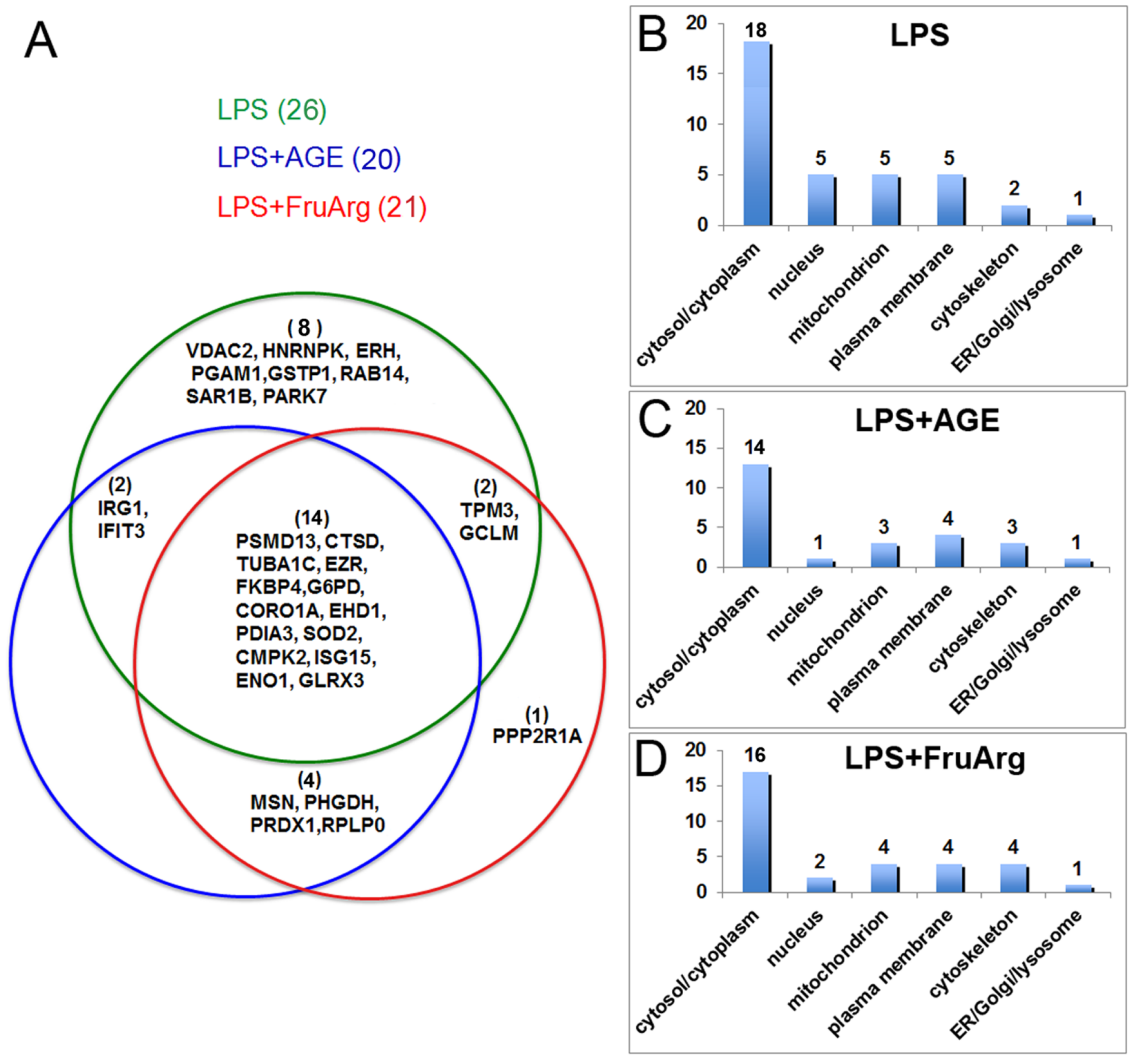
Data analysis for the identified differentially expressed proteins using MULTICOM-PDCN. (**A**) Three subsets of differential expression proteins with overlaps in between were identified from different treatments (LPS, LPS+AGE, and LPS+FruArg) in BV-2 cells; Cellular locations of these proteins after treated with LPS (**B**), treated with LPS in the presence of AGE (**C**) or FruArg (**D**) were predicted using the in-house MULTICOM-PDCN software.

In this study, a total of 26, 20, and 21 differentially expressed proteins were identified in response to LPS, LPS+AGE, and LPS+FruArg treatments, respectively (Figure 6A). Among these, LPS+AGE treatment shared 16 differentially expressed proteins with LPS-alone treatment; and LPS+FruArg treatment elicited changes in 16 proteins in common with LPS treatment, thus indicating both AGE and FruArg partially attenuated the effects of LPS in BV-2 cells. Eighteen proteins responded to the treatments with both AGE and FruArg, accounting for approximate 78% overlap. Nevertheless, AGE and FruArg treatment also had distinct targets. For the LPS+AGE treatment, 2 unique proteins, while for the LPS+FruArg treatment, 3 proteins were uniquely altered, respectively. Taken together, these findings suggest FruArg as a major bioactive component of AGE, and a potential modulator of oxidative and nitrosative stress and neuroinflammation.

Among those identified proteins with the differential expression levels, cellular component analysis using MULTICOM-PDCN located 14 in cytosol/cytoplasm, 1 in nucleus, 3 in mitochondrion, 4 in plasma membrane, 3 in cytoskeleton, and 1 in ER/Golgi/lysosome responding to AGE (Figure 6B), as well as 16, 2, 4, 4, and 4 in those organelles, respectively, in response to FruArg. These results indicate both AGE and FruArg regulate majority of proteins located in cytosol/cytoplasm. Importantly, the nuclear compartment appeared to contain the highest proportion of LPS-modulated proteins whose expression was altered by treatment with AGE or FruArg (5 versus 1 and 2, respectively), suggesting that AGE and FruArg share a common mechanism of transcriptional regulation of protein levels.

### Effects of LPS in BV-2 cells on canonical pathways and signaling networks

**Figure 7:**
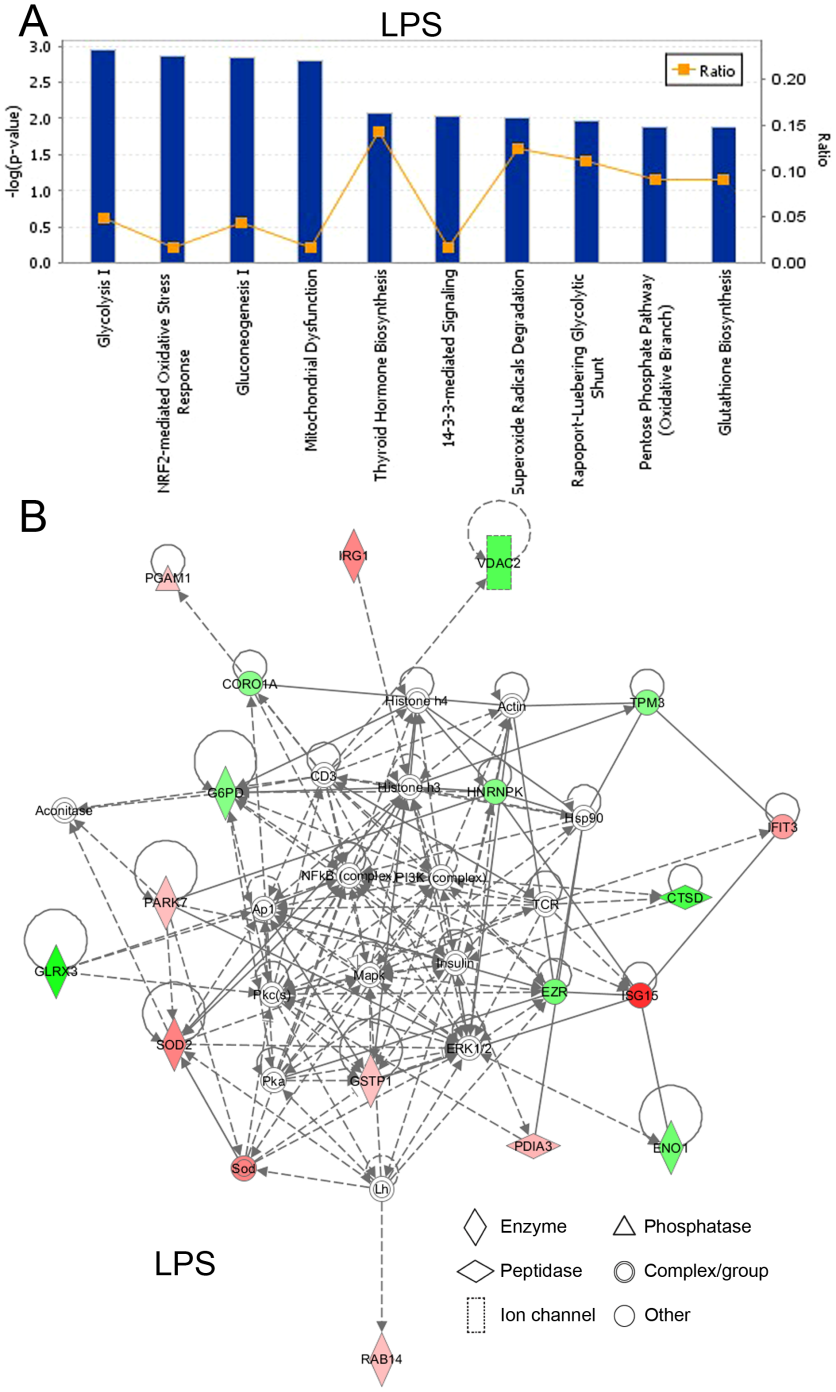
Effects of LPS in BV-2 cells on canonical pathways and signaling networks. (**A**) Top 10 canonical pathways participated by the differentially expressed proteins responding to LPS treatment in BV-2 cells. The canonical pathways were ranked according to their −log (p-value). A ratio (orange square) indicates the number of identified differentially proteins found in each pathway over the total number of proteins in that pathway. (**B**) The top protein-protein interaction network responding to LPS treatment in BV-2 cells is mainly associated with drug metabolism, protein synthesis, DNA replication, recombination, and repair. The symbols labeled in red and green represent up- and down-regulation, respectively, and the intensity of the colors indicates the degree of regulation. Solid lines in the network imply direct interactions between proteins, and dashed lines indicate indirect interactions.

IPA annotation indicated that the 26 differentially expressed proteins responding to LPS treatment in BV-2 cells play critical roles in inflammatory response (13 proteins, p value at 4.31E-05—2.90E-02) and neurological disease (12 proteins, p value at 1.16E-05—3.03E-02), with participation in various cellular processes including drug metabolism (8 proteins, p value at 2.11E-07—3.02E-02), protein synthesis (14 proteins, p value at 2.11E-07—3.02E-02), DNA replication, recombination, and repair (6 proteins, p value at 9.10E-08—2.42E-02), and cell death and survival (17 proteins, p value at 8.22E-07—2.98E-02). The top canonical pathways for the proteins altered by LPS according to the order of the −log (p value) include glycolysis I, Nrf2-mediated oxidative stress response, gluconeogenesis I, mitochondrial dysfunction, thyroid hormone biosynthesis, 14-3-3-mediated signaling, and superoxide radicals degradation (Figure 7A). Protein-protein interaction networks were further examined by IPA analysis. Results showed that the top protein-protein interaction network (Figure 7B) is mainly associated with drug metabolism, protein synthesis, DNA replication, recombination, and repair (score = 49). Eighteen out of the 26 identified proteins responding to LPS treatment are involved in this network. Among these proteins, 9 proteins are down-regulated (shaded green) and 9 proteins were up-regulated (shaded red).

### Effects of AGE and FruArg in LPS-stimulated BV-2 cells on canonical pathways

**Figure 8:**
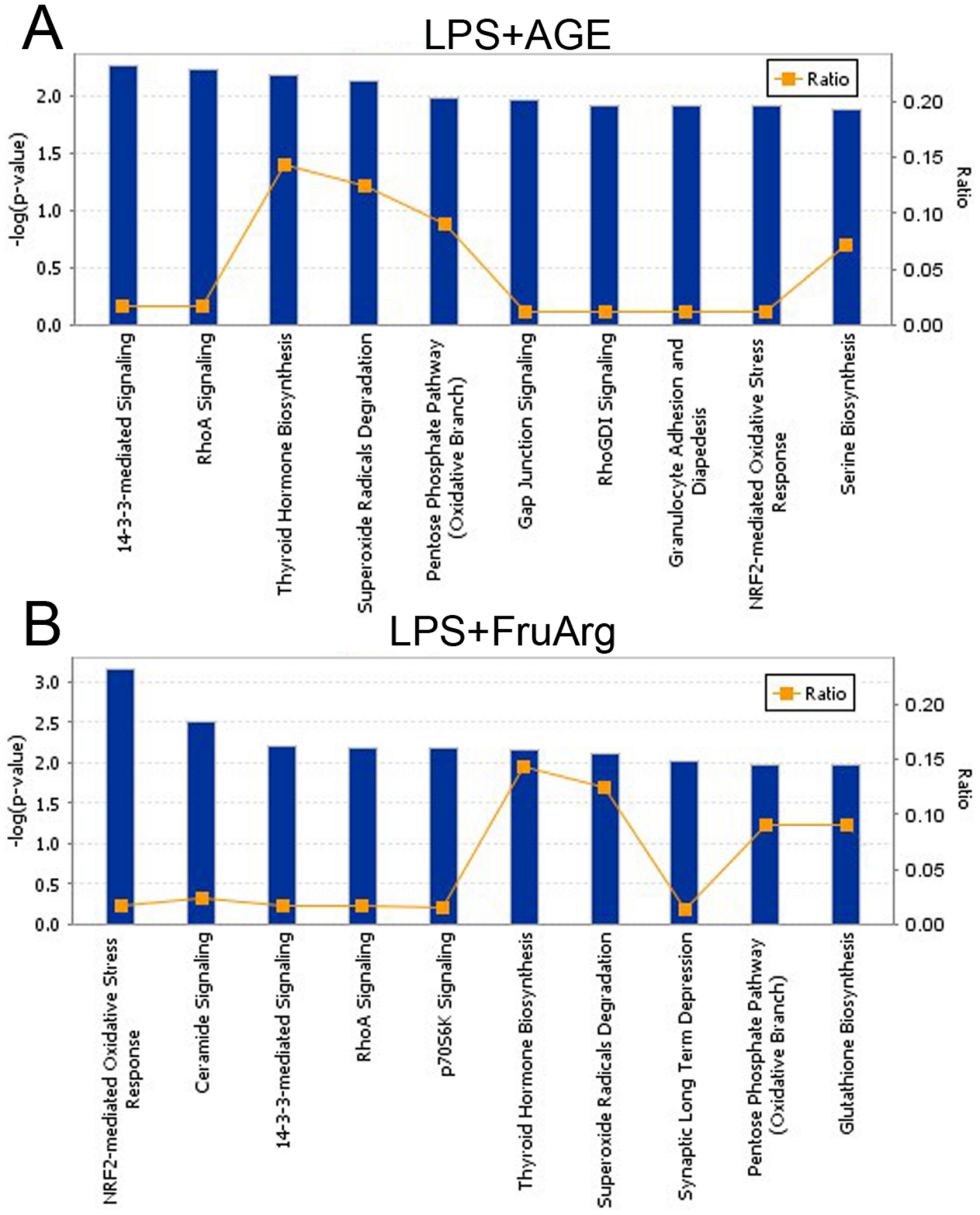
Canonical pathways altered by AGE and FruArg in LPS-stimulated BV-2 cells. IPA analysis using the differentially expressed proteins predicted the canonical pathways targeted by AGE (**A**) or FruArg (**B**) in LPS-stimulated BV-2 cells. Of the top 10 canonical pathways, 6 pathways are shared by the two botanical compounds. These canonical pathways were ranked according to their −log (p-value).

In order to gain more insight into the mechanisms for the action of AGE and FruArg in LPS-induced microglial activation, we performed pathway and functional analyses of the differentially expressed proteins. IPA annotation indicates that the proteins sensitive to the AGE and FruArg treatments are associated with immunological disease and inflammatory response, and function in cell death and survival, free radical scavenging, DNA replication, recombination, and repair, cellular assembly and organization (p<0.05). Results of the canonical pathway analysis suggested Nrf2-mediated oxidative stress response, 14-3-3-mediated signaling, RhoA signaling, thyroid hormone biosynthesis, superoxide radicals degradation, and pentose phosphate pathway (oxidative branch) were among the top canonical pathways targeted by treatment of the two botanical compounds (Figure 8). In comparison, AGE uniquely attenuated effect of LPS on gap junction signaling, RhoGDI signaling, granulocyte adhesion and diapedesis, and serine biosynthesis (Figure 8A), whereas FruArg specifically affected ceramide signaling, p70S6K signaling, synaptic long term depression, and glutathione biosynthesis (Figure 8B). Among the top 10 canonical pathways targeted by LPS (Figure 7A), there are five shared pathways also modulated by AGE or FruArg (Figure 8A and 8B). Hence, AGE and FruArg may reverse the LPS-induced alteration of these key pathways involved in regulation of cellular redox status, cell cycle control, inflammatory signaling and stress responses, biosynthesis and energy metabolism.

### Signaling networks affected by AGE and FruArg in LPS-stimulated BV-2 cells

**Figure 9:**
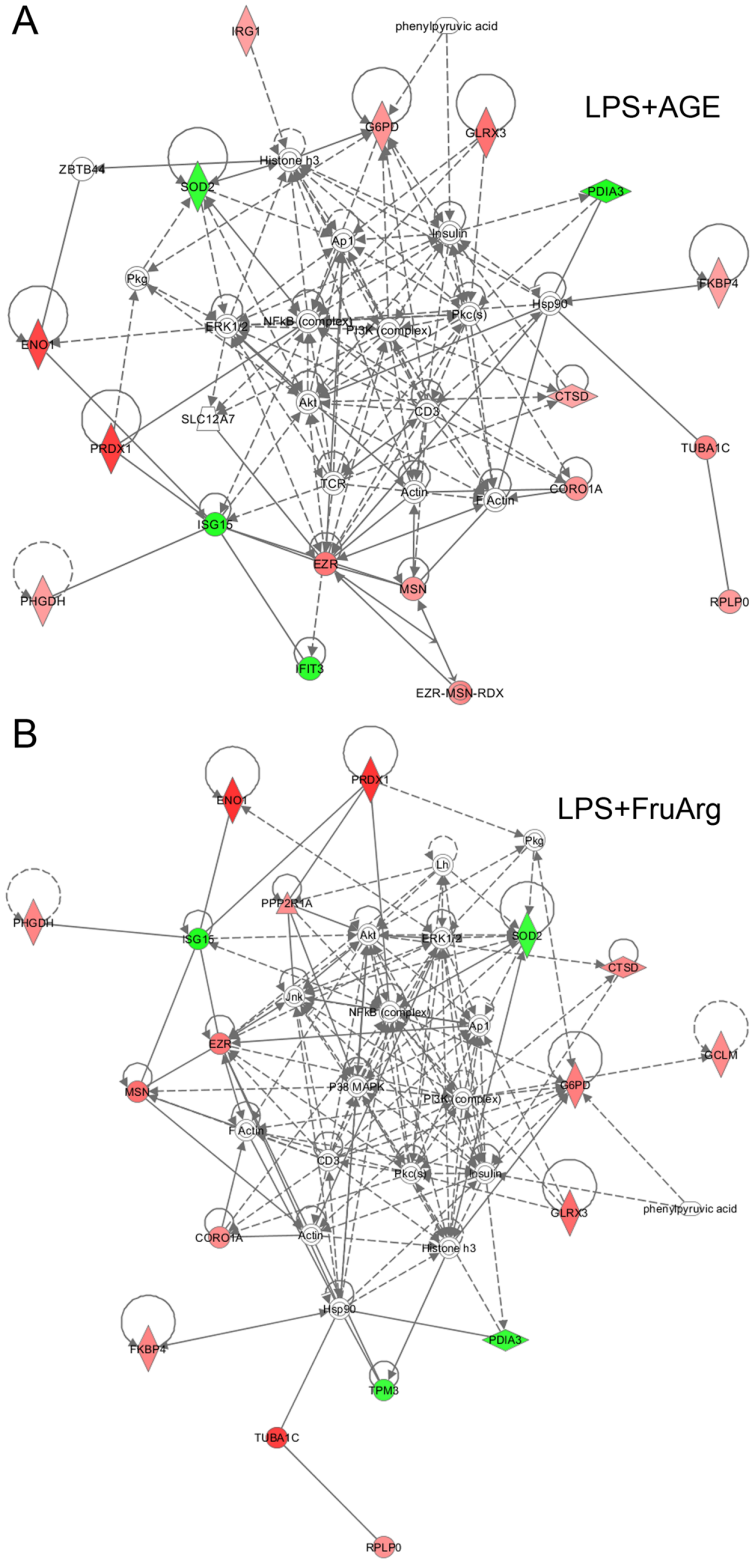
Protein-protein interaction networks associated with AGE and FruArg treatments in LPS-stimulated BV-2 cells. The networks analysis result showed AGE modulates protein networks that function in immunological disease and cellular assembly and organization (**A**), while FruArg modulates protein networks that are mainly associated with immunological disease, drug metabolism, and cellular assembly and organization (**B**). There were 15 common proteins shared by the two protein networks.

We further examined the effects of AGE and FruArg on protein-protein interaction networks in LPS-stimulated BV-2 cells. Based on IPA analysis, the top-score protein-protein interaction network associated with AGE treatment functions in immunological disease and cellular assembly and organization (IPA score = 49). Eighteen out of the 20 identified proteins responding to AGE treatment are involved in this network (Figure 9A). Among those proteins, 4 proteins were down-regulated (shaded green) and 14 proteins were up-regulated (shaded red). In the case of FruArg treatment, the top protein-protein interaction network functions in immunological disease, drug metabolism, and cellular assembly and organization (IPA score = 52), consisting of 18 of the 21 identified proteins (Figure 9B). Among those proteins, 4 proteins were down-regulated (shaded green) and 14 proteins were up-regulated (shaded red). There are 15 common proteins (CORO1A, CTSD, EZR, FKBP4, G6PD, GLRX3, ISG15, MSN, PDIA3, PHGDH, PRDX1, SOD2, ENO1, TUBA1C, RPLP0) between these two networks, further confirming that FruArg serves as a major functional component in AGE.

## Discussion

Chronic activation of microglia can lead to inflammatory responses through release of various inflammatory mediators that increase oxidative stress, trigger neuronal cell death, and have been implied in the development of neurodegenerative diseases [31],[40]. In this study, we showed that LPS-induced microglial activation, a well-established cellular model of neuroinflammation, altered expression levels of a number of proteins (such as up-regulation of PDIA3, PARK7 and SOD2), consistent with previous reports showing that LPS activates microglia to release neurotoxic factors to induce neuronal death [31],[41]. The 26 proteins responding to LPS-stimulation are linked to drug metabolism, protein synthesis, DNA replication, recombination, and repair (Figure 7B). IPA annotation of canonical pathways showed that Nrf2-mediated pathway, a major antioxidant mechanism for neuroprotection against oxidative stress [42],[43], is one of the top targets under LPS exposure (Figure 7A).

AGE, an odorless substance prepared by aging garlic for up to 20 months, has been shown to have neuroprotective effects upon cerebral oxidative stress and neuronal cell death in experimental models of neurodegenerative disorders including cerebral ischemia and Alzheimer's disease [44]–[47]. Furthermore, different molecular and cellular mechanisms have been reported, such as scavenging reactive oxygen species [48],[49], inhibition of pro-oxidant enzymes [46],[50], and induction of antioxidant enzymes [51],[52]. FruArg, a product of the Maillard reaction during the aging process of AGE, has been reported to be capable of inducing both anti-oxidative and anti-inflammatory effects [16],[53]. However, much remains unknown about the intracellular targets and molecular mechanism(s) underlying the effects of these botanicals. In this study using 2D-DIGE analyses, we have demonstrated that AGE or FruArg treatment can significantly decrease nitrosative stress, as well as regulate the expression of multiple proteins, thus posing a multi-modal action of AGE and FruArg in activated microglia.

2D-DIGE quantitative proteomics analysis showed that treatment with AGE and FruArg to LPS-stimulated BV-2 cells resulted in alteration of 20 and 21 proteins, respectively (Figure 6A). The regulation effects of LPS on 18 proteins (about 69% of the total 26 identified proteins) were reversed by AGE or FruArg treatment. Interestingly, treatment with AGE or FruArg decreased more proteins compared to that of the BV-2 cells exposed to LPS alone in the compartment of nucleus (5 proteins versus 1 and 2, respectively). These results imply that, similarly to a number of anti-inflammatory botanicals, AGE and FruArg may modulate toll-like receptors (TLRs)-induced activation and nuclear translocation of proinflammatory transcription factors for gene regulation [54]–[56].

As a result, the modulation of LPS in 5 top canonical pathways, including Nrf2-mediated oxidative stress response, thyroid hormone biosynthesis, 14-3-3-mediated signaling, superoxide radicals degradation, and pentose phosphate pathway (oxidative branch) was restored by AGE and FruArg (Figure 7A and Figure 8). In particular, both AGE and FruArg altered the Nrf2-mediated signaling pathway, which is a chief mechanism for cellular defense against oxidative stress [57]. These results suggest that AGE and FruArg attenuated LPS-induced neuroinflammatory responses in BV-2 cells through regulating expression of proteins that are involved in multiple oxidative stress-related pathways, consistent with their known antioxidant effects.

Additionally, 78% of the identified proteins that responded to AGE and FruArg treatments were found in common (Figure 6A). And not surprisingly, six of the top ten canonical pathways predicted by IPA are shared by the two botanical compounds (Figure 8). These results indicate that FruArg is a major bioactive component of AGE.

Previous studies demonstrated the average concentration of FruArg in various AGE preparations to be 2.2±0.2 mM [11], which is close to the concentration used for FruArg treatment (3 mM) here. Thus, we considered this dose to be the human consumption equivalent. However, for AGE treatment, we made a 200-fold dilution, since other active compounds possess synergistic antioxidant properties and most likely also contribute to its effect on suppression of nitrosative stress and neuroinflammatory responses. Nevertheless, we observed the ability for FruArg as well as AGE to inhibit LPS-induced NO production in microglial cells. Similar results for AGE were found in LPS-stimulated rat macrophages [58], suggesting that AGE and FruArg may be considered as promising dietary supplements to promote resilience in nitrosative stress-induced neurodegenerative disorders by suppressing production of proinflammatory molecules and promoting antioxidant pathways.

## Conclusions

In this study, we used gel-based quantitative proteomics to investigate the effects of AGE and FruArg in LPS-stimulated BV-2 cells and revealed new mechanistic insights into the multiple protein targets of these botanical compounds to mitigate neuroinflammatory responses. In addition, our results demonstrated FruArg as a major active component of AGE and similarly acting on nitrosative stress and neuroinflammation. Based on the expression levels of the identified proteins, we predicted multiple signal transduction pathways associated with these targets. Evidence supporting the multi-modal action of AGE and FruArg may aid in better understanding of the molecular mechanisms for their promotion of resilience against the effects of neurological diseases and aging.
